# Bosutinib reduces endothelial permeability and organ failure in a rat polytrauma transfusion model^1^

**DOI:** 10.1016/j.bja.2021.01.032

**Published:** 2021-03-06

**Authors:** Derek J.B. Kleinveld, Liza Botros, M. Adrie W. Maas, Jesper Kers, Jurjan Aman, Markus W. Hollmann, Nicole P. Juffermans

**Affiliations:** 1 Department of Intensive Care Medicine, Amsterdam UMC, University of Amsterdam, Amsterdam, The Netherlands; 2 Laboratory of Experimental Intensive Care and Anesthesiology, Department of Trauma Surgery, Amsterdam UMC, University of Amsterdam, Amsterdam, The Netherlands; 3 Department of Trauma Surgery, Amsterdam UMC, University of Amsterdam, Amsterdam, The Netherlands; 4 Department of Pulmonary Diseases, Amsterdam UMC, Vrije Universiteit Amsterdam, Amsterdam, The Netherlands; 5 Department of Physiology, Amsterdam UMC, Vrije Universiteit Amsterdam, Amsterdam, The Netherlands; 6 Department of Pathology, Amsterdam Infection & Immunity Institute, Amsterdam Cardiovascular Sciences, Amsterdam UMC, University of Amsterdam, Amsterdam, The Netherlands; 7 Department of Pathology, Leiden University Medical Center, University of Leiden, Leiden, The Netherlands; 8 Van't Hoff Institute for Molecular Sciences (HIMS), University of Amsterdam, Amsterdam, The Netherlands; 9 Ragon Institute of Massachusetts General Hospital, Massachusetts Institute of Technology and Harvard University, Cambridge, MA, USA; 10 Department of Anesthesiology, Amsterdam UMC, University of Amsterdam, Amsterdam, The Netherlands; 11 Department of Intensive Care Medicine, Onze Lieve Vrouwe Gasthuis, Amsterdam, The Netherlands

**Keywords:** bosutinib, endothelial dysfunction, shock, transfusion, trauma, tyrosine kinase inhibitor

## Abstract

**Background:**

Trauma-induced shock is associated with endothelial dysfunction. We examined whether the tyrosine kinase inhibitor bosutinib as an adjunct therapy to a balanced blood component resuscitation strategy reduces trauma-induced endothelial permeability, thereby improving shock reversal and limiting transfusion requirements and organ failure in a rat polytrauma transfusion model.

**Methods:**

Male Sprague–Dawley rats (*n*=13 per group) were traumatised and exsanguinated until a MAP of 40 mm Hg was reached, then randomised to two groups: red blood cells, plasma and platelets in a 1:1:1 ratio with either bosutinib or vehicle. Controls were randomised to sham (median laparotomy, no trauma) with bosutinib or vehicle. Organs were harvested for histology and wet/dry (W/D) weight ratio.

**Results:**

Traumatic injury resulted in shock, with higher lactate levels compared with controls. In trauma-induced shock, the resuscitation volume needed to obtain a MAP of 60 mm Hg was lower in bosutinib-treated animals (2.8 [2.7–3.2] ml kg^−1^) compared with vehicle (6.1 [5.1–7.2] ml kg^−1^, *P*<0.001). Lactate levels in the bosutinib group were 2.9 [1.7–4.8] mM compared with 6.2 [3.1–14.1] mM in the vehicle group (*P*=0.06). Bosutinib compared with vehicle reduced lung vascular leakage (W/D ratio of 5.1 [4.6–5.3] *vs* 5.7 [5.4–6.0] (*P*=0.046) and lung injury scores (*P*=0.027).

**Conclusions:**

Bosutinib as an adjunct therapy to a balanced transfusion strategy reduced resuscitation volume, improved shock reversal, and reduced vascular leak and organ injury in a rat polytrauma model.

**Editor's key points**

• Trauma-induced shock is associated with endothelial dysfunction, which is reduced by a balanced blood component resuscitation strategy.
• The protective effect of the tyrosine kinase inhibitor bosutinib was tested in a rat polytrauma model because inhibitors protect endothelial integrity in models of vascular leak.
• Bosutinib-treated rats required less resuscitation volume and had reduced lung vascular leakage and lung injury scores.
• Bosutinib provide a potential adjunct to a balanced transfusion strategy in protecting organ function in the treatment of traumatic shock if validated in human studies.

Use of a balanced 1:1:1 red blood cell/plasma/platelet transfusion ratio has improved outcome of traumatic bleeding^1^; however, morbidity and mortality remain high.^2^ Research efforts in trauma have focused on treating or preventing trauma-induced coagulopathy, resulting in shifts towards mortality later in the disease process during intensive care.^1^^,^^3^, ^4^, ^5^ Whereas early mortality is predominantly attributed to exsanguination and traumatic brain injury, late mortality is caused by organ failure.^6^^,^^7^ Organ failure is thought to be mediated by inflammation-induced endothelial activation, resulting in leakage and oedema.^6^^,^^8^ Adjunctive treatment strategies aimed at limiting organ failure in trauma by minimising dysfunctional endothelial barrier function have not been thoroughly investigated.^9^

Tyrosine kinase inhibitors, such as bosutinib, are used in the treatment of chronic myeloid leukemia.^10^, ^11^, ^12^ Some of these inhibitors have been shown to protect endothelial integrity in a mouse model of vascular leakage*.*^13^ Bosutinib compared with other tyrosine kinase inhibitors was superior in protecting against inflammation-induced vascular leakage.^14^ The mechanism of bosutinib-induced reduction in vascular leakages involves combined inhibition of the Abl-related gene (Arg) kinase and mitogen activated protein 4 kinase 4 (MAP4K4), which results in reinforcement of endothelial cell–cell junctions and enhanced adhesion to the subcellular matrix.^14^^,^^15^ In animal models, other tyrosine kinase inhibitors have shown benefits on endothelial function in the settings of acute lung injury,^16^^,^^17^ cardiopulmonary bypass,^18^ and in a haemodilution shock model.^19^ However, none of these studies evaluated bosutinib in an acute setting along with a balanced transfusion resuscitation strategy.

The aim of this study was to investigate the effect of bosutinib as an adjunctive therapy to a balanced transfusion strategy against endothelial leakage, resuscitation volume needed to restore perfusion, and organ failure in a rat model of multiple trauma and transfusion. We hypothesised that bosutinib limits endothelial leakage leading to a reduction in transfusion requirements to restore perfusion, thereby reducing organ injury.

## Methods

### Animals

The experiments described in this study were approved by the Institutional Animal Care and Use Committee of the Amsterdam University Medical Centers. The procedures were performed in accordance with the European Parliament directive (2010/63/EU) and the national law of the Experiments on Animals Act (Wod, 2014). A total of 52 male Sprague–Dawley rats weighing 350–400 g (Envigo, Indianapolis, IN, USA) were used. In addition, 40 male rats were used for preparation of blood products. Animals were group housed with access to water and food *ad libitum* at least 7 days before the experiment. Housing of animals was temperature-controlled with a 12/12 h light/dark cycle.

### Transfusion products

Transfusion products were made from syngeneic donor rats according to national blood bank standards as described.^20^

### Multiple trauma transfusion model

Anaesthesia was induced with ketamine 100 mg kg^−1^ (Alfasan BV, Woerden, The Netherlands), dexmedetomidine 0.25 mg kg^−1^ (Orion Pharma, Espoo, Finland) and atropine 0.1 mg kg^−1^ (Dechra, Bladel, The Netherlands) i.p. and maintained with a combination of ketamine 33.3 mg kg^−1^ h^−1^ and dexmedetomidine 0.02 mg kg^−1^ h^−1^ i.v. Rats were tracheotomised and mechanically ventilated using a pressure control mode (Babylog 8000; Draeger, Lubeck, Germany) with a ventilatory frequency of 60 min^−1^ (70 min^−1^ during shock), PEEP of 3.5 kPa, and inspiratory pressures (*P*_insp_) of 10 kPa. Tidal volumes were maintained between 6 and 7 ml kg^−1^ in all groups. Recruitment was performed every hour by increasing *P*_insp_ to 25 kPa for 5 s. Blood pressure was measured continuously via a cannula (fine bore polyethylene tubing, inner diameter 0.58 mm) inserted in the right carotid artery. The right jugular vein was cannulated for administration of fluids and the allocated treatment. Maintenance fluids consisted of Ringer's lactate at 4 ml kg^−1^ h^−1^. The bladder was emptied before trauma, and urine output was measured during the experiment. Temperature was maintained at 36.5–37.0°C using a heat table and heat lamp.

**Supplementary Fig S1:**
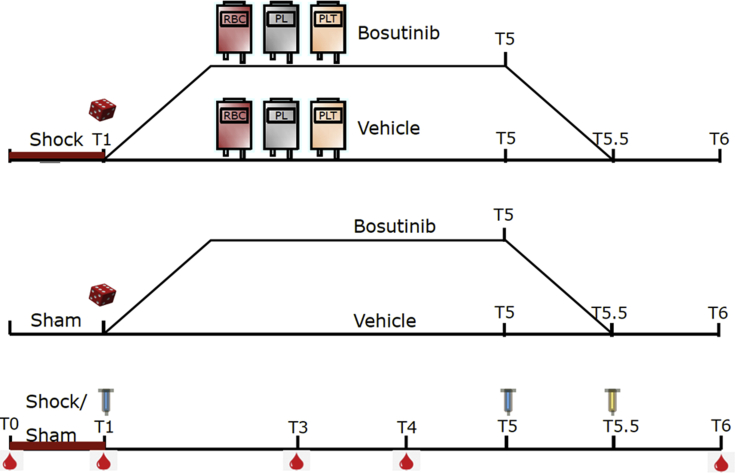
Schematic overview of the trauma transfusion model. Overview of the trauma transfusion model. T0 = baseline sample, T1 = 1 hour after injury and end of shock period, moment of randomization, initiation of treatment strategy, T3 = post-transfusion sample, T4 = additional blood gas sample to adjust ventilation if necessary, T5 = extra dose of bosutinib or vehicle if randomized to one of these groups, T5.5 administration of 70kDa FITC-labelled dextran, and T6 = 6 h after injury, termination of the animal by blood draws and harvesting of different organs. RBC = red blood cell, PL = plasma product, PLT = platelets, RL = Ringer’s lactate (crystalloid).

Trauma was induced by a fracture of the right femur using a guillotine, a crush injury to the small intestine and left lateral liver lobe as described^20^ (Supplementary Fig. S1). Then, an initial volume of blood was drawn from the carotid artery according to the following formula (0.30[0.06 × weight + 0.77]),^21^ and further adjusted in steps of 0.5 ml to reach a MAP of <40 mm Hg (total initial volume drawn: 30–40% of circulating volume). In contrast to a previous model,^20^ the duration of shock was 1 h, after which rats were randomised to two groups: resuscitation with either a balanced 1:1:1 ratio of red blood cells/plasma/platelets either with bosutinib (selleckchem.com; Pfizer, New York, NY, USA; 5 mg kg^−1^ dissolved in 2% DMSO/30% PEG/5% Tween-20 in a volume of 200 μl NaCl 0.9%) or with vehicle initially given as a bolus before transfusion. The operator was blinded to treatment allocation. Transfusion was administered at a speed of 8 ml h^−1^ (Perfusor® fm; B Braun, West Bloomfield Township, MI, USA) in the jugular vein. After an animal reached a MAP of 60 mm Hg lasting for at least 5 min, transfusion was stopped. The concentration of bosutinib was based on a previous experiment.^22^ Owing to the half-life of bosutinib, a repeated dose of bosutinib or vehicle was given 5 h after trauma or sham.^23^

Control groups were randomised separately to sham with bosutinib or vehicle. Sham consisted of a median laparotomy without inflicting traumatic injury. The laparotomy was closed immediately in order to avoid hypothermia. Bosutinib or vehicle was administered directly after shock or at a similar time in the sham groups.^23^

### Measurements

Blood was drawn before trauma (*T*=0); after trauma-induced shock and before initiation of the resuscitation strategy (*T*=1 h); and 3, 4, and 6 h after trauma (*T*=3, 4, and 6). Arterial blood gas analyses were performed at all time points, rotational thromboelastometry (ROTEM) at two time points (T0, T6), and biochemical assessments at two time points (T0, T6). Plasma was prepared and stored at –80°C after double spinning (20 min, 2500 *g*, 18°C, acceleration 9, brake 0; 5804 R, Eppendorf AG, Hamburg, Germany).

Syndecan-1 (Elabscience, Houston, TX, USA), soluble vascular adhesion molecule-1 (VCAM-1; Elabscience), tumour necrosis factor-α and interleukin-6 (IL-6; R&D Systems, Minneapolis, MN, USA) were determined with enzyme-linked immunosorbent assays according to manufacturer guidelines. The lowest detection limit was noted when values were below the detection limit. Aspartate and alanine transaminases, creatinine, and total urine protein were measured by standard enzymatic reactions using spectrophotometric, colorimetric or turbidimetric measurement methods. The estimated glomerular filtration rate was calculated as: (urine creatinine at termination × urine production in 6 h)/plasma creatinine.

To assess vascular leakage, 0.5 ml of 12.5 mg ml^−1^ fluorescein isothiocyanate (FITC)-labelled 70 kDa dextran (Sigma Aldrich, St. Louis, MO, USA) was injected in the jugular vein 30 min before animal sacrifice. Rats were sacrificed by exsanguination after which organs were flushed with 50 ml NaCl 0.9% administered via the left side of the heart whereas the right jugular catheter was connected to the flushing system using gravity. Before flushing, the hilum of the left lung and left kidney were tied off to assess these organs for wet/dry (W/D) ratio.

All organs were assessed by haematoxylin and eosin staining by a pathologist, who was blinded for treatment allocation.^20^^,^^24^ The scale of each category consisted of a score of 0 (absent) to 3 (severe). For the full scoring list, see Supplementary Table S1.

#### Immunohistochemical analysis of vascular leakage

Slides of organs were deparaffinised and coloured using a rabbit–anti-FITC/anti-rabbit horseradish peroxidase (HRP) and NovaRed colouring method. The staining time was kept constant within each organ system, ranging from 5 min (lung) to 7 min (small intestine) per slide. Five different images of each slide were obtained with a microscope (10× magnification) and a camera (BX51 with UC90; Olympus Corp., Tokyo, Japan). Pictures were inverted using Image J (1.50i, NIH, www.imagej.net). Five random inverted pictures were used to set a threshold for FITC-70 kDa dextran leakage. The median percentage of the area intensity was used as measure of endothelial leakage.

#### Outcomes

The primary outcome was the transfusion volume until a MAP of 60 mm Hg was reached for 5 min. The secondary outcomes were circulating markers of endothelial damage and inflammation, vascular leakage, organ oedema, and organ failure parameters.

### Statistical analyses

#### Sample size analysis

Based on previous experiments,^20^ we estimated a treatment effect of 0.6 ml reduction in transfusion volume with a standard deviation (sd) of 0.4 ml. For this scenario, 11 rats per group provide a power of 80% to detect a statistically significant difference in transfusion volume needed to reach a MAP of 60 mm Hg using a Mann–Whitney *U*-test with a two-sided significance level of *P*<0.05. As we previously experienced a mortality of ∼20% in our model, we added two additional rats per group (*n*=13 per group).

#### Data analysis

Data were analysed using SPSS (version 25.0; IBM Corp., Chicago, IL, USA), graphs were made using GraphPad Prism (version 8.0.2; GraphPad Software Inc., San Diego, CA, USA). All parameters were checked for normality using a Kolmogorov–Smirnov test and by visual inspection of histograms. Data were presented as mean (sd) or median (inter-quartile range [IQR]) based on their distribution. The transfusion volume needed to reach a MAP of 60 mm Hg was evaluated with a Mann–Whitney *U*-test. Other outcomes were tested for differences using Kruskal–Wallis test. *Post hoc* tests were performed between sham and trauma groups and between trauma + vehicle and trauma + bosutinib groups. A *P* value <0.05 was considered statistically significant.

## Results

### Shock, transfusion needs, and mortality

**Fig 1:**
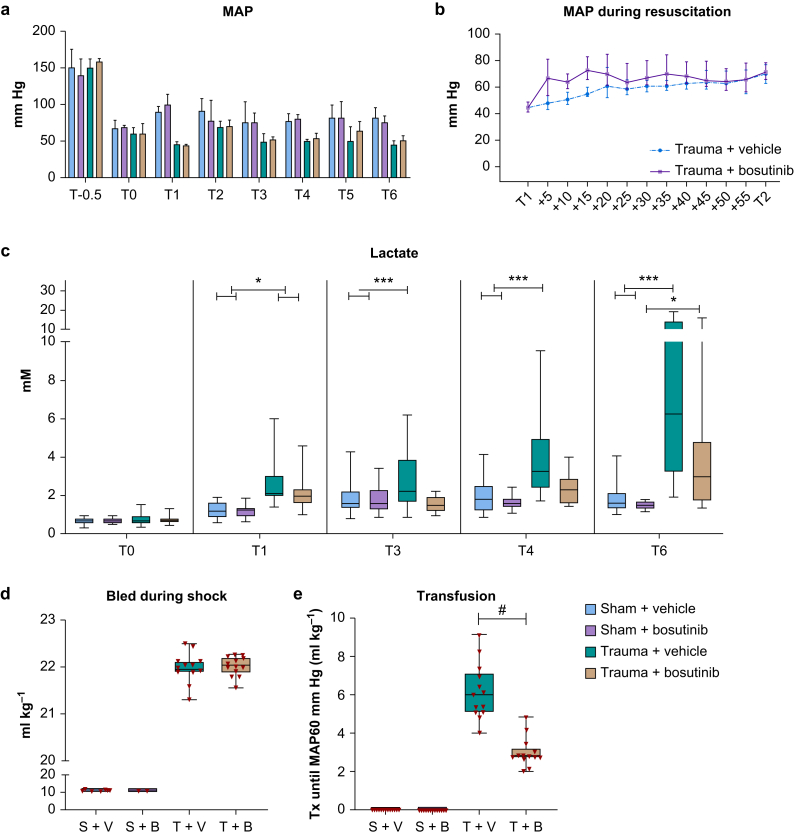
Bosutinib improved shock reversal and reduced resuscitation volume. Data are median (inter-quartile range). T–0.5 (before trauma), T0 (trauma), T1 (start resuscitation), T2–6=2 to 6 h after trauma. (a) Mean arterial pressure (MAP). Between T–0.5 and T0, traumatic injury was initiated, the first blood draw was used as baseline sample. Injury and traumatic shock lasted for 1 h, thereafter rats were randomised to receive allocated transfusion and treatment (Tx), controls were sham with bosutinib or vehicle. (b) Mean arterial pressure for trauma groups during resuscitation (T1–T2). (c) Lactate levels. (d) Volume of haemorrhage during shock phase. (e) Volume of transfusion needed to restore an MAP of 60 mm Hg. In traumatic injury, the vehicle and bosutinib groups were treated with standard transfusion strategy of a 1:1:1 red blood cell/plasma/platelet ratio. S+V, sham + vehicle; S+B, sham + bosutinib; T+V, trauma + vehicle; T+B, trauma + bosutinib. Sham *vs* trauma groups: ∗*P*<0.05, ∗∗*P*<0.01. Trauma + vehicle vs trauma + bosutinib: ^#^*P*<0.05.

There was no mortality in the experimental and control groups. Trauma and the shock phase resulted in shock, with increased base deficit compared with sham groups (Table 1 and Fig. 1). No differences in arterial blood gas analysis were found between trauma groups after shock (Table 1). After resuscitation (*T*=3), median lactate levels in the bosutinib group (1.5 [1.2–2.0] mM) were not significantly different compared with the vehicle group (2.2 [1.6–3.9] mM; *P*=0.06) (Fig. 1). This trend toward lower lactate levels in the bosutinib group (2.9 [1.7–4.8] mM) compared with the vehicle group (6.2 [3.1–14.1] mM; *P*=0.06) remained until termination (*T*=6). Animals in the bosutinib group required lower transfusion volume for shock reversal (2.8 [2.7–3.2] ml kg^−1^ in 7.5 [7.5–9.0] min) compared with vehicle (6.1 [5.1–7.2] ml kg^−1^ in 15.8 [15.0–19.5] min; *P*<0.001) (Fig. 1). Quality assessments of the different products are described in Supplementary Table S2.

**Table 1:** Baseline and trauma-induced shock parameters.

Parameter	Sham + vehicle		Sham + bosutinib		Trauma + vehicle		Trauma + bosutinib	
	Baseline	1 h (T1)	Baseline	1 h (T1)	Before injury	After injury and shock (*T*=1)	Before injury	After injury and shock (*T*=1)
Weight (g)	365 (7)	ND	363 (12)	ND	368 (19)	ND	366 (13)	ND
Total blood volume (ml)	22.7 (0.4)	ND	22.0 (0.7)	ND	22.9 (1.1)	ND	22.7 (0.8)	ND
Temperature (°C)	36.6 (36.5–37.0)	36.4 (36.3–36.7)	36.7 (36.6–36.9)	36.5 (36.4–36.7)	36.4 (36.3–36.8)	36.5 (36.2–36.9)	36.7 (36.4–36.9)	36.5 (36.1–36.8)
pH	7.43 (7.36–7.47)	7.37 (7.33–7.43)	7.41 (7.38–7.44)	7.38 (7.35–7.40)∗	7.40 (7.39–7.45)	7.34 (7.31–7.36)∗	7.43 (7.40–7.46)	7.34 (7.31–7.38)∗
pCO_2_ (kPa)	5.1 (4.6–5.9)	5.1 (4.3–5.9)	5.3 (4.5–6.0)	5.3 (4.7–5.8)	4.9 (4.6–5.3)	5.1 (4.7–5.4)	5.0 (4.3–5.2)	4.8 (4.7–6..0)
BE (mEq L^−1^)	0.3 (–1.5 to 1.1)	–2.8 (–3.3 to –1.3)	0.6 (0.2–2.1)	–1.8 (–3.2 to –0.7)	–0.7 (–2.3 to 0.4)	–5.1 (–7.3 to –4.5)∗	–0.3 (–1.2 to 0.5)	–4.1 (–5.2 to –3.6)∗
Lactate (mM)	0.7 (0.6–0.9)	1.2 (1.0–1.2)	0.6 (0.5–0.7)	1.3 (0.9–1.4)	0.6 (0.5–1.0)	2.1 (1.9–3.0)∗	0.6 (0.6–0.8)	2.0 (1.6–2.3)∗
Hb (mM)	9.1 (8.4–9.3)	8.0 (7.6–8.4)	9.4 (8.8–9.6)	8.0 (7.5–8.5)	9.3 (8.9–9.6)	7.4 (7.0–8.2)	9.1 (8.9–9.3)	7.8 (7.0–8.2)
Ca^2+^ (mM)	1.2 (1.1–1.2)	1.1 (1.0–1.2)	1.2 (1.0–1.2)	1.3 (1.2–1.3)	1.1 (1.0–1.2)	1.1 (0.9–1.1)∗	1.1 (1.0–1.2)	1.1 (0.9–1.2)∗
Glucose (mM)	20.4 (18.6–21.4)	27.8 (25.4–30.2)	22.7 (21.5–23.1)	27.0 (25.4–28.2)	23.9 (19.3–26.2)	38.2 (34.1–40.7)∗	22.6 (20.7–24.2)	36.5 (34.5–38.2)∗

Data are presented as mean (standard deviation) or median (inter-quartile range). BE, base excess; Hb, haemoglobin; ND, no data. ∗*P*<0.05 compared with sham groups. No significant differences were found between the trauma + vehicle and trauma + bosutinib groups.

### Coagulation

Normal ROTEM values were observed in sham groups. The EXTEM and FIBTEM maximum clot firmness (MCF) was significantly different between the sham groups and the trauma + vehicle group. Despite the lower transfusion volume in the trauma + bosutinib compared with vehicle groups, no differences were found in ROTEM and platelet counts between groups (Table 2).

**Table 2:** Endotheliopathy, inflammation, and laboratory parameters of organ injury.

Parameter	Sham + vehicle		Sham + bosutinib		Trauma + vehicle		Trauma + bosutinib	
	Baseline	After 6 h	Baseline	After 6 h	Baseline	After 6 h	Baseline	After 6 h
*Endotheliopathy*								
sVCAM-1 (pg ml^−1^)	283 (265–382)	289 (77–502)	265 (209–307)	200.0 (141–285)	244 (204–279)	818 (524–2183)∗	273 (238–339)	352 (269–650)^#^
Syndecan-1 (ng ml^−1^)	31.0 (27.0–35.1)	61.3 (55.0–69.0)	31.2 (27.0–33.8)	46.6 (30.1–74.0)	32.3 (27.5–37.4)	110.8 (43.7–142.6)∗	33.3 (26.7–39.3)	51.2 (37.2–71.6)
Hepatic								
AST (U L^−1^)	64.0 (60.0–68.0)	181.0 (105.0–356.0)	65.5 (60.3–69.8)	149.0 (121.0–195.5)	67.0 (59.5–69.0)	2670 (2047.5–4806.5)∗∗	66.0 (61.0–68.0)	1574.0 (579.5–2686.5)∗∗
ALT (U L^−1^)	48.0 (45.5–55.5)	63.0 (49.5–107.5)	54.0 (50.5–57.0)	63.0 (47.5–91.5)	57.0 (48.0–62.0)	1719.0 (1326.0–3034.0)∗∗	52.0 (47.5–60.0)	798.5 (254.5–1468.5)∗∗
*Renal*								
Creatinine (μM)	24.0 (22.5–26.5)	62.0 (43.0–91.5)	23.5 (22.3–25.0)	50.0 (41.0–62.0)∗∗	25.0 (24.0–29.0)	139.5 (115.8–163.5)∗∗	26.0 (25.0–28.5)	120.5 (93.5–141.8)∗
Urine protein (g L^−1^)	0.5 (0.2–0.9)	1.0 (0.6–1.5)	0.6 (0.2–0.8)	0.8 (0.7–1.4)	0.6 (0.3–1.0)	1.7 (1.5–2.1)∗	0.5 (0.4–0.8)	1.6 (0.8–2.0)
Urine output (ml kg^−1^)	ND	11.4 (9.5–13.3)	ND	14.5 (10.1–19.9)	ND	7.4 (4.2–9.7)∗∗	ND	10.9 (8.7–13.3)^#^
eGFR (ml min^−1^)	ND	1.11 (0.44–1.51)	ND	1.16 (0.50–1.63)	ND	0.11 (0.04–0.21)∗∗	ND	0.25 (0.15–0.56)∗∗
*Inflammation*								
Leucocytes (×10^9^ L^−1^)	5.8 (4.8–6.5)	7.0 (4.9–8.6)	6.6 (5.8–8.0)	8.3 (6.5–8.9)	6.3 (5.2–7.5)	5.2 (4.5–7.8)	6.7 (5.0–7.6)	6.2 (4.5–7.3)
^±^IL-6 (pg ml^−1^)	<12	<12	<12	<12	<12	1639 (707–2526)∗∗	<12.0	121 (12–1020)∗^, #^
^±^TNF-α (pg ml^−1^)	<62.5	<62.5	<62.5	<62.5	<62.5	<62.5	<62.5	<62.5
*Coagulation*								
Platelets (×10^9^ L^−1^)	881 (819–916)	564 (504–600)	947 (780–1014)	582 (552–628)	970 (934–1019)	479 (345–593)	903 (889–964)	573 (506–650)
EXTEM CT (s)	52 (49–55)^#^	51 (45–57)	49 (46–53)	49 (47–58)	47 (46–50)	53 (49–74)	48 (47–51)	45 (43–51)
EXTEM MCF (mm)	69 (69–72)	71 (69–71)	71 (70–73)	70 (70–73)	71 (70–74)	67 (62–69)∗	71 (70–72)	70 (66–70
EXTEM Li30 (%)	100 (100–100)	100 (100–100)	100 (100–100)	100 (100–100)	100 (100–100)	100 (100–100)	100 (100–100)	100 (100–100)
FIBTEM CT (s)	49 (48–51)	49 (42–51)	46 (44–50)	46 (43–51)	43 (41–47)	54 (47–69)	42 (39–44)	43 (40–49)
FIBTEM MCF (mm)	14 (14–15)	13 (13–14)	15 (14–15)	13 (12–14)	15 (14–19)	10 (6–13)∗	15 (13–15)	11 (9–13)

Data are presented as median (inter-quartile range). Before trauma/sham and at termination, samples were measured for different parameters. ALT, alanine aminotransferase; AST, aspartate aminotransferase; eGFR, estimated glomerular filtration rate determined by urine creatinine times urine production in 6 h divided by plasma creatinine; IL-6, interleukin 6; ND, not determined; ROTEM EXTEM, rotational thromboelastometry; sVCAM-1, soluble vascular adhesion molecule 1; TNF-α, tumour necrosis factor alpha. ±values below detection limit are depicted as lower than the lowest reference range. ∗*P*<0.05, ∗∗*P*<0.01 sham *vs* trauma groups, ^#^*P*<0.05, ^##^*P*<0.01 trauma group + vehicle *vs* trauma + bosutinib group.

### Systemic organ dysfunction and inflammation

Animals with trauma-induced shock developed severe organ damage compared with sham animals as shown by increased creatinine and liver transaminase levels (Table 2), which were not different between trauma groups. Bosutinib treatment resulted in higher urine output compared with vehicle, and IL-6 levels were lower in the bosutinib-treated rats compared with vehicle-treated rats (Table 2).

### Endothelial damage and leakage

**Fig 2:**
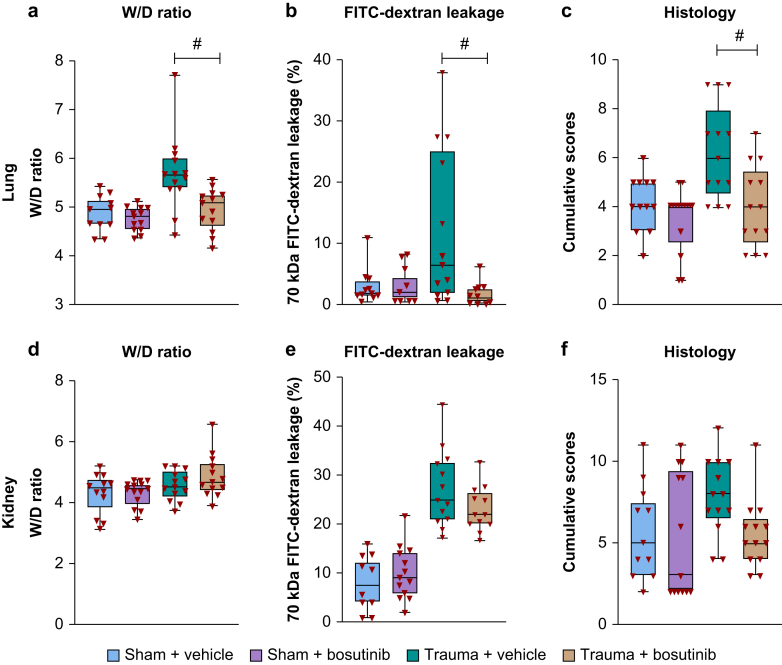
Bosutinib reduced organ oedema, vascular leakage and organ injury score in lung and kidney. Data are median (inter-quartile range) or boxplot with range. (a–c) Lung WD, lung FITC-70 kDa dextran leakage and total lung score. (d–f) Kidney WD, kidney FITC-70 kDa dextran leakage and total kidney score. Significance is shown for trauma + vehicle vs trauma + bosutinib. ^#^*P*<0.05. W/D, wet/dry; FITC, fluorescein isothiocyanate.

Endothelial damage identified by elevated plasma syndecan-1 and soluble vascular cell adhesion molecule-1 (sVCAM-1) was present in all trauma-induced shock groups. Bosutinib treatment resulted in reduced plasma levels of sVCAM-1 compared with vehicle (Table 2). Also, bosutinib-treated rats showed lower lung W/D ratios (5.1 [4.6–5.3]) compared with vehicle (5.7 [5.4–6.0]; *P*=0.046) (Fig. 2) and less dextran leakage in lung (1% [0–3%] *vs* 6% [2–25%]; *P*=0.01). However, bosutinib did not limit vascular leakage of dextran in other organs (liver, spleen, small intestine, kidney).

### Organ damage and mortality

**Fig 3:**
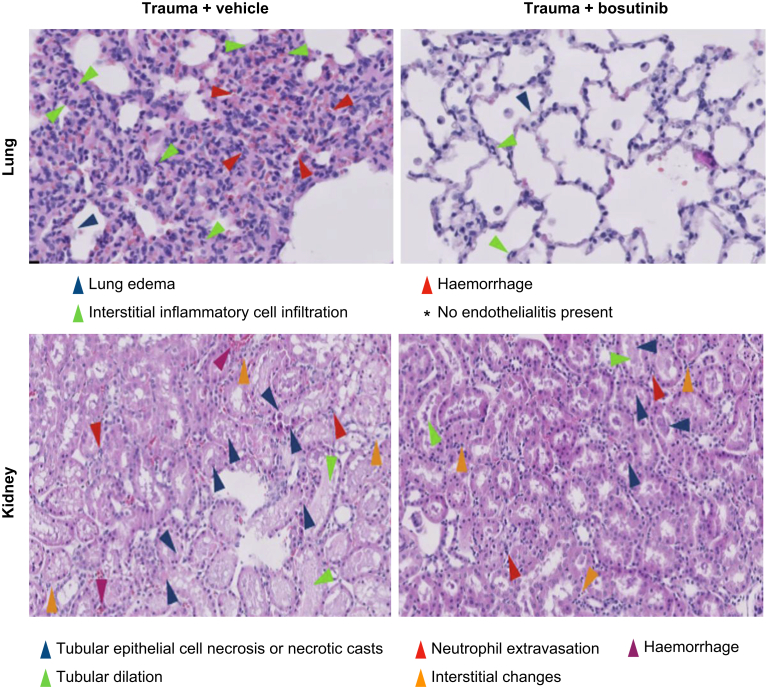
Histology of lung and kidney after trauma-induced shock.

**Supplementary Fig S2:**
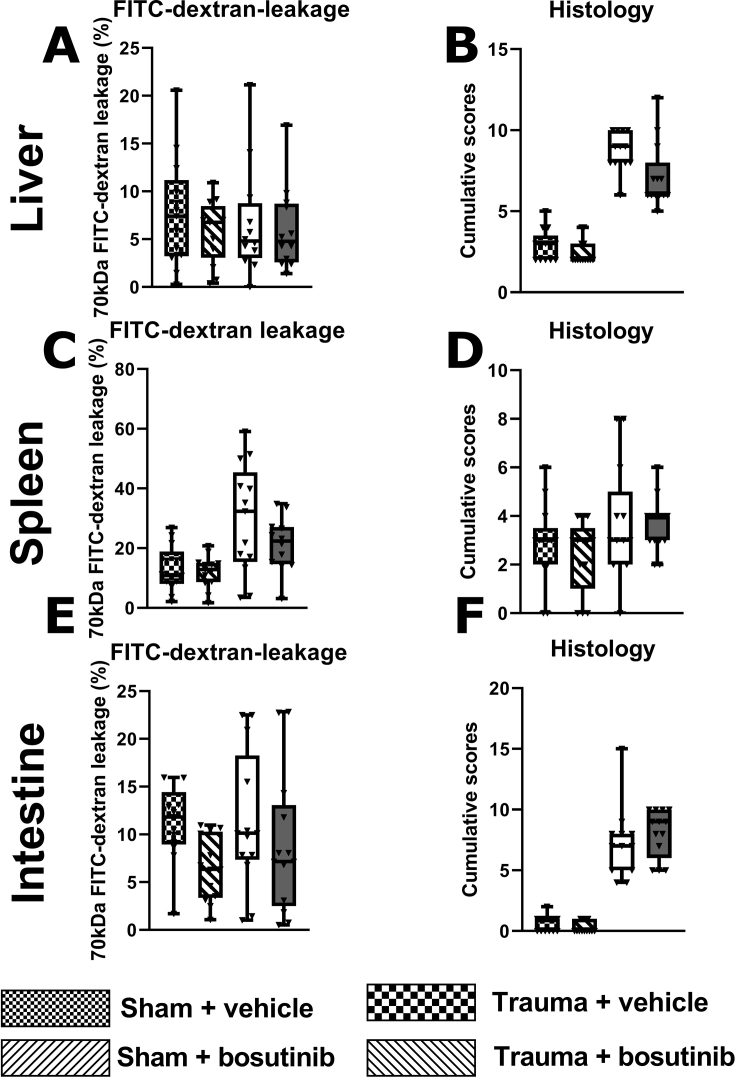
Bosutinib has limited effects on injury of the splanchnic organs. Data are median (IQR) or boxplot with range. (a, b) Liver FITC-70 kDa dextran leakage and liver score. C – D Spleen FITC-70 kDa dextran leakage and spleen score. E – F Small intestine FITC-70 kDa dextran leakage and small intestine score.

**Supplementary Fig S3:**
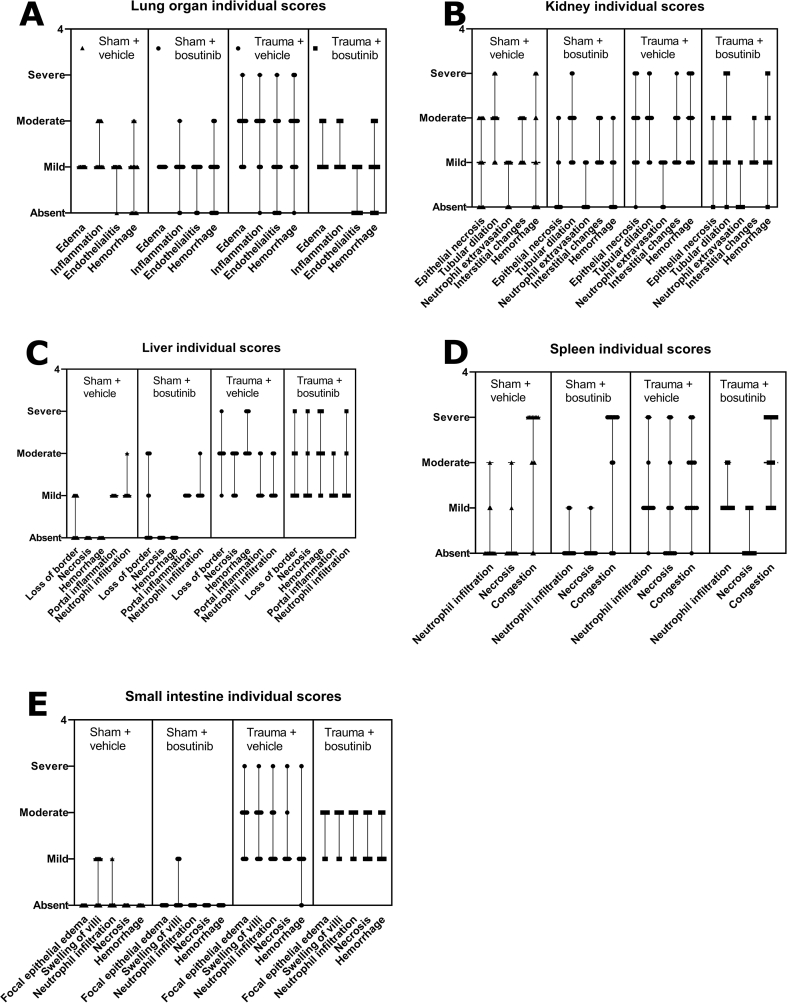
Individual organ scores. Data are median with range (all points are shown).

Bosutinib treatment resulted in lower total lung organ injury (4.0 [2.5–5.5]) compared with vehicle (6.0 [4.5–8.0]; *P*=0.027) (Fig 2, Fig 3), but did not affect organ injury scores of kidney, liver, spleen, and small intestine (Fig 2, Fig 3 and Supplementary Fig. S2). The distributions of individual organ injury scores are shown in Supplementary Figure S3. No mortality was observed.

## Discussion

Bosutinib as adjunctive therapy to a balanced transfusion strategy was associated with reduced transfusion requirement, improved shock reversal, and reduced endothelial leakage with concomitant reduction in pulmonary oedema and lung injury when compared with a vehicle-treated control group. Bosutinib-treated rats also had less evidence of endothelial damage. Collectively, these data suggest that bosutinib can protect endothelial barrier integrity in traumatic bleeding, and can reduce transfusion requirements to restore circulation in a trauma model.

Our findings of improved shock reversal, endothelial barrier function, and organ protective effects of bosutinib are in line with previous studies, in which bosutinib reduced lipopolysaccharide-induced lung permeability.^14^ Other tyrosine kinase inhibitors such as imatinib reduce organ oedema in several disease models, including acute lung injury, sepsis, cardiopulmonary bypass, and trauma and hypovolaemic shock models.^16^^,^^18^^,^^19^^,^^25^^,^^26^ We extend these findings by showing efficacy in a polytrauma haemorrhagic shock model with a balanced transfusion resuscitation strategy. We used bosutinib instead of imatinib as bosutinib has a stronger effect on endothelial tight junctions and hence barrier integrity compared with imatinib, and because bosutinib inhibits both Arg and MAP4K4 kinases whereas imatinib has a high specificity for Arg kinase.^14^ The effects of bosutinib on organ injury were most clear for lung. As lung injury is often the first manifestation of a multiple organ dysfunction syndrome, lung protection may result in better outcomes.^27^^,^^28^ However, the long-term effects of bosutinib in trauma-induced shock remain to be determined.

IL-6 levels were lower in bosutinib-compared with vehicle-treated rats with traumatic injury. The immunomodulatory effects of bosutinib have been shown in mouse-derived macrophages, in which bosutinib reduced IL-6 production.^29^ Less endothelial damage might lead to a reduced inflammatory response as well.

The bosutinib-treated group required less transfusion products (and hence less plasma and platelets) than the vehicle group, whereas coagulation parameters in the ROTEM assays did not differ. In addition to an effect on endothelial barrier function, bosutinib may also reduce endothelial-driven coagulopathy in this model. Further evaluation is necessary to pinpoint the effects of bosutinib on trauma-induced coagulopathy, including platelet function and coagulation factors.

Both the reduced endothelial leakage and transfusion volume required to restore circulation by bosutinib could have contributed to organ protection,^6^, ^7^, ^8^ as both endothelial leakage and use of transfusion products have been associated with organ failure in trauma.^6^^,^^30^^,^^31^ We chose a resuscitation strategy targeted at a predetermined blood pressure. A model with a fixed transfusion volume with a primary outcome of organ failure might have identified which factor contributed more to reduced organ failure. However, this may not be a clinically relevant question because a fixed volume approach can induce overtransfusion.

Because bosutinib i.v. has rapid bioavailability *in vivo*,^32^ is clinically licensed, and has a better safety profile compared with other tyrosine kinase inhibitors,^15^ administration of bosutinib in a bleeding trauma patient is feasible. With regard to safety, there are concerns about decreasing platelet counts and low-grade liver toxicity in patients on long-term treatment with tyrosine kinase inhibitors.^11^^,^^15^^,^^33^^,^^34^ However, in an acute setting in which only one or two doses are to be administered, the protective effects of bosutinib may outweigh potential side-effects. Moreover, we did not observe differences in platelet counts or liver injury when comparing sham animal treated with bosutinib to sham animals treated with vehicle.

This study has certain limitations. Despite a prior power analysis, the sample size was probably too small to reveal differences in kidney injury scores. However, all effects of bosutinib were in the direction of organ protection. Because many secondary outcomes were exploratory, we did not correct for multiple testing. The rats used in this model were young and male; it remains to be determined whether the endothelial response to bosutinib is influenced by age, sex, or vascular comorbidities. Furthermore, the coagulation system in rats differs from that in humans.^35^^,^^36^ For example rats have 3- to 4-fold higher platelet counts than humans. The effects of bosutinib should be further studied in trauma models with longer durations of mechanical ventilation to determine its long-term side-effects and benefits in an acute setting.

In conclusion, bosutinib reduced resuscitation needs, vascular leakage, and lung injury in a trauma and transfusion model. Although preliminary, these findings suggest that bosutinib may be an appealing therapeutic option to ameliorate organ failure in severe traumatic bleeding patients. Further study is required to determine whether it can improve outcomes after trauma.

## Authors’ contributions

Design: DJBK, LB, JA, MWH, NPJ.

Data collection: DJBK, MAWM, JK.

Statistical analysis: DJBK, NPJ.

Drafting of the manuscript: DJBK, NPJ.

Revision and final approval of the manuscript: all authors.

## Declarations of interest

MWH is Executive Section Editor Pharmacology with Anesthesiology and Section Editor Anesthesiology with the *Journal of Clinical Medicine*. He has received research funding from ZonMW, STW, SCA, ESA, Eurocept BV, Edwards Life Sciences. MWH served as consultant for Eurocept BV and ECHO BV and received speaker fees from CSL Behring and BBraun. NPJ has received research support from CSL Behring, Octapharma and Werfen and research funding from H2020, Haemonetics, ZonMW, Sanquin, ESICM, and the Dutch military blood bank. All other authors have declared no conflicts of interest.

## Funding

Institutional resources.

## Appendix A Supplementary data

Multimedia component 1 Supplementary Table S1. Organ failure assessment score. All evaluations were made on five fields per section and five sections per organ. Severity was assessed based on a scale from 0 to 3 (0, absent; 1, mild; 2, moderate; 3, severe). Supplementary Table S2 Quality assessment of blood products. In total 13 different red blood cell, plasma products and platelet products were made. Measurements are on the day of transfusion. Some measurements had missing values because of technical errors in the blood gas analysis equipment or cell counter. Maximum missing values were 6/13 products. ND=not determined. NA=not applicable. Multimedia component 1
