# Supplementary material for: Bosutinib reduces endothelial permeability and organ failure in a rat polytrauma transfusion model
Source: Br J Anaesth. 2021 Mar 6;126(5):958–66. doi: 10.1016/j.bja.2021.01.032 (PMC8258973; doi:10.1016/j.bja.2021.01.032)
Supplement: Multimedia component 1 — Supplementary Table S1. Organ failure assessment score. All evaluations were made on five fields per section and five sections per organ. Severity was assessed based on a scale from 0 to 3 (0, absent; 1, mild; 2, moderate; 3, severe). Supplementary Table S2 Quality assessment of blood products. In total 13 different red blood cell, plasma products and platelet products were made. Measurements are on the day of transfusion. Some measurements had missing values because of technical errors in the blood gas analysis equipment or cell counter. Maximum missing values were 6/13 products. ND=not determined. NA=not applicable. [file mmc1.docx]

**Supplemental table legends**

**Supplemental table 1: Organ failure assessment score**

| **Organ failure assessment score** |
| --- |
| **Lung injury** was scored as follows: |
| - Lung edema - Interstitial inflammatory cell infiltration - Endothelialitis - Hemorrhage |
| The severity of **liver injury** was scored as follows: |
| - Loss of intercellular borders - Necrosis - Hemorrhage - Portal inflammation - Neutrophil infiltration |
| The severity of **spleen injury** was scored as follows: |
| - Neutrophil infiltration - Necrosis - Congestion |
| The severity of **kidney injury** was scored as follows: |
| - Tubules in the cortex or the outer medulla that showed epithelial necrosis or had luminal necrotic debris - Tubular dilation - Neutrophil extravasation - Interstitial changes - Hemorrhage |
| The severity of **small intestine injury** was scored as follows: |
| - Focal epithelial edema - Diffuse swelling of the villi - Neutrophil infiltration in the submucosa - Necrosis - Hemorrhage |

All evaluations were made on five fields per section and five sections per organ. Severity was assessed based on a scale from 0-3 (0, absent; 1, mild; 2, moderate; 3, severe).

| Parameter | **Red blood cell product** | | **Plasma product** | | **Platelet product** | |
| --- | --- | --- | --- | --- | --- | --- |
|  | Trauma + vehicle | Trauma + bosutinib | Trauma + vehicle | Trauma + bosutinib | Trauma + vehicle | Trauma + bosutinib |
| pH | 7.0 (7.0 – 7.1) | 7.1 (7.0 – 7.2) | 7.2 (7.1 – 7.2) | 7.2 (7.1 – 7.2) | 7.1 (7.0 – 7.1) | 7.1 (7.0 – 7.1) |
| Lactate (mmol/L) | 1.5 (1.3 – 2.4) | 1.4 (1.0 – 1.6) | 1.7 (1.2 – 2.2) | 1.3 (1.1 – 2.3) | 2.5 (1.9 – 2.7) | 2.5 (2.0 – 3.2) |
| Hb (mmol/L) | 12.3 (10.4 – 12.7) | 12.2 (10.1 – 12.9) | NA | NA | NA | NA |
| K^+^ (mmol/L) | 1.5 (1.3 – 2.9) | 1.6 (1.1 -1.6) | 3.1 (3.1 – 3.2) | 3.1 (2.9 – 3.2) | 3.3 (3.2 – 3.5) | 3.2 (3.2 – 3.4) |
| Platelets (*10^9/L) | ND | ND | <100 | <100 | 1184 (1112 – 1496) | 1092 (1071 – 1496) |
| Leukocytes ((*10^9/L) | 1.6 (1.6 – 2.7) | 1.8 (1.4 – 2.5) | NA | NA | NA | NA |

**Supplemental table 2: Quality assessment of blood products**

In total 13 different red blood cell, plasma products and platelet products were made. Measurements are on the day of transfusion. Some measurements had missing values due to technical errors in the blood gas analysis equipment or cell coulter. Maximum missing values were 6/13 products. ND=not determined. NA=not applicable.
